# To: Severe hypercalcemia as a form of acute lymphoblastic leukemia presentation in children

**DOI:** 10.5935/0103-507X.20160034

**Published:** 2016

**Authors:** José Colleti Junior, Werther Brunow de Carvalho

**Affiliations:** 1 Pediatric Intensive Care Unit, Hospital Santa Catarina - São Paulo (SP), Brazil.; 2 Department of Pediatrics, Instituto da Criança, Universidade de São Paulo - São Paulo (SP), Brazil.

**To the Editor,**

**Figure 1:**
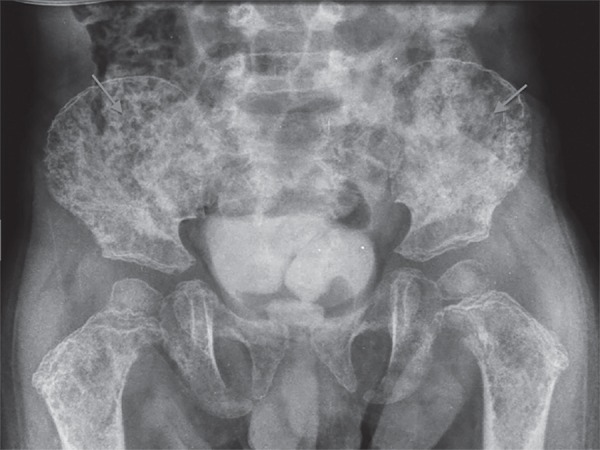
Pelvic X-ray showing osteolytic lesions (arrows).

**Figure 2:**
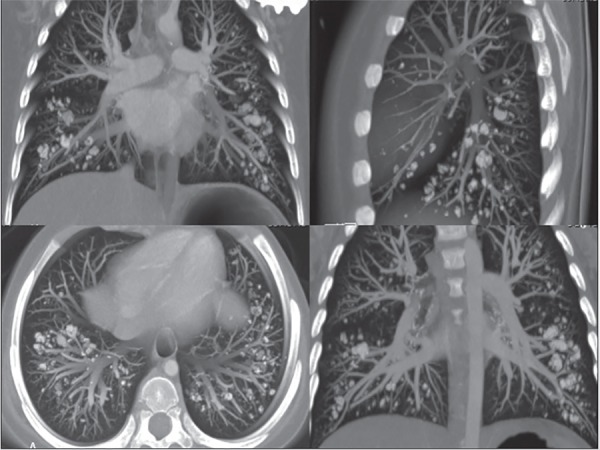
Computed chest tomography showing alveolar calcification.

Severe hypercalcemia of malignancy in children has been extensively described in the medical literature.^(1)^ However, this complication usually presents as a late symptom of acute lymphoblastic disease and not as an early manifestation, as described in a case report by Martins et al.^(2)^ Colleti Junior et al.^(3)^ also observed that hypercalcemia was present as an early symptom of malignancy, and the serum calcium levels were higher than usual (ionic calcium: 2.95mmol/L; normal value: 1.11-1.40mmol/L). In this case, immediately after diagnosis, in addition to hydration and low doses of loop diuretic, treatment was initiated with pamidronate, a bisphosphonate, and resulted in a rapid decrease in the serum calcium levels. In the case described, it is unclear whether hemofiltration was performed to purify the calcium or to treat kidney failure; the latter does not seem to be the case considering the initial test results. In the first case, early treatment with bisphosphonates might have avoided an invasive procedure, such as hemofiltration.^(3,4)^ It is important to describe the protein levels of parathyroid hormone (PTHrP) - often reported as normal - because, in most cases, hypercalcemia caused by malignant disease is associated with the production of PTHrP,^(5)^ and the measurement of this protein is essential for the differential etiological diagnosis. In addition, the absence of signs of osteopenia is noteworthy in this case because the high calcium levels are due in part to osteolytic activity, which is blocked by bisphosphonate (otherwise, the calcium levels would not have decreased with the use of zoledronate). Colleti Junior et al.^(3)^ reported that the osteolytic activity is so extensive that classic lesions are observed in a punch biopsy (Figure 1). Another relevant aspect is the evaluation of tissue calcium deposition after treatment with bisphosphonates. Is there a report of nephrocalcinosis related to calcium deposition? Is there a report of calcium deposition in other tissues? In Colleti Junior et al.,^(3)^ a rare alveolar lung deposition was shown on chest computed tomography (Figure 2), and the diagnosis of this symptom was essential during patient follow-up. Therefore, the side effects of treatment with bisphosphonates should be considered.

Furthermore, we highlight the study of the authors and make a note to pediatricians about this important clinical sign and the need for monitoring treatment-related aspects.

José Colleti Junior

Pediatric Intensive Care Unit, Hospital Santa Catarina - São Paulo (SP), Brazil.

Werther Brunow de Carvalho

Department of Pediatrics, Instituto da Criança, Universidade de São Paulo - São Paulo (SP), Brazil.
